# An EM Induction Hi-Speed Rotation Angular Rate Sensor

**DOI:** 10.3390/s17030610

**Published:** 2017-03-17

**Authors:** Kai Li, Yuan Li, Yan Han

**Affiliations:** 1 Institute of Information Sensing and Processing Technology, North University of China, Taiyuan 030051, China; hanyan@nuc.edu.cn; 2 School of Information and Communication Engineering, North University of China, Taiyuan 030051, China; liyuan82@nuc.edu.cn

**Keywords:** sensor, angular rate, high rotational speed, EM induction

## Abstract

A hi-speed rotation angular rate sensor based on an electromagnetic induction signal is proposed to provide a possibility of wide range measurement of high angular rates. An angular rate sensor is designed that works on the principle of electromagnetism (EM) induction. In addition to a zero-phase detection technique, this sensor uses the feedback principle of magnetic induction coils in response to a rotating magnetic field. It solves the challenge of designing an angular rate sensor that is suitable for both low and high rotating rates. The sensor was examined for angular rate measurement accuracy in simulation tests using a rotary table. The results show that it is capable of measuring angular rates ranging from 1 rps to 100 rps, with an error within 1.8‰ of the full scale (FS). The proposed sensor is suitable to measurement applications where the rotation angular rate is widely varied, and it contributes to design technology advancements of real-time sensors measuring angular acceleration, angular rate, and angular displacement of hi-speed rotary objects.

## 1. Introduction

Angular rate sensors are important elements for measuring flying motion parameters. They find extensive applications in consumer electronics, industries, and armament sectors.

An angular rate sensor can be implemented by use of optical, mechanical, or electromagnetic effects, different physical effects producing different types of sensor. Angular rate sensors are either internal or external, depending on where they are installed. Internal ones are largely implemented using an inertia sensor [1], a mechanical rotor [2], or magnetic field effect [3,4,5] technology, whilst external ones generally use image analysis [6,7] or mechanical gear methods. Internal ones, such as MEMS [8,9], optical gyroscopes [10], and electrostatic gyroscopes [11,12], are subject to limited room, impact influence, and EM interference and thus are limited in both sensing accuracy and measurement range. External sensors, tachometers for example, are capable of high accuracy and large range measurement within their detection range but are unable to measure the angular rate of a body tracked at a long range.

In industrial applications, a flying body generally involves a spinning or rotation angular rate between 2 rps and 50 rps. A sensor operating on the principle of an EM field effect provides a solution to the problems of measurement range and accuracy. Additionally, for detecting the attitude of a spinning projectile, a magnetometer can be incorporated to estimate the projectile body roll and pitch angles [13]. Existing magnetic sensors measure angular rate generally by measuring the angular rate of hi-speed spinning projectiles by use of a magnetic reluctance sensor and by studying the time-frequency characteristics of the magnetic signal [14], by building a multi-functional sensor, or a new measurement system, that combines the characteristics of a magnetic sensor and another type of sensor [15,16], and by using an electrostatic effect-based gyroscope, which is effective in measuring angular rates below 50 rps [17,18], in tracking mechanical frequency modulation input, and in performing signal frequency analysis and making up a high-frequency vibration angular rate sensor [19,20,21].

A novel sensor based on magnetic induction is proposed in this paper for measuring angular rates. This is an internal sensor by design and is useful for measuring angular rates varying from 1 to 100 rps. The layout of magnetic induction coils is described, their magnetic induction physical response characteristics are analyzed, and a model is constructed that describes the feedback of the magnetic induction sensor in response to a rotating carrier magnetic field. The sensor signal is then analyzed using an electronic solution technique. Tests involving a low- and a hi-speed rotary table were conducted to evaluate the sensor performance at an angular rate of 1 rps and 100 rps. The measurement accuracy is 0.1‰–1.8‰ FS, validating the sensor performance.

## 2. Design Principle of the Rotating Body Angular Rate Sensor 

### 2.1. Coil Layout of the Magnetic Induction Sensor

**Figure 1:**
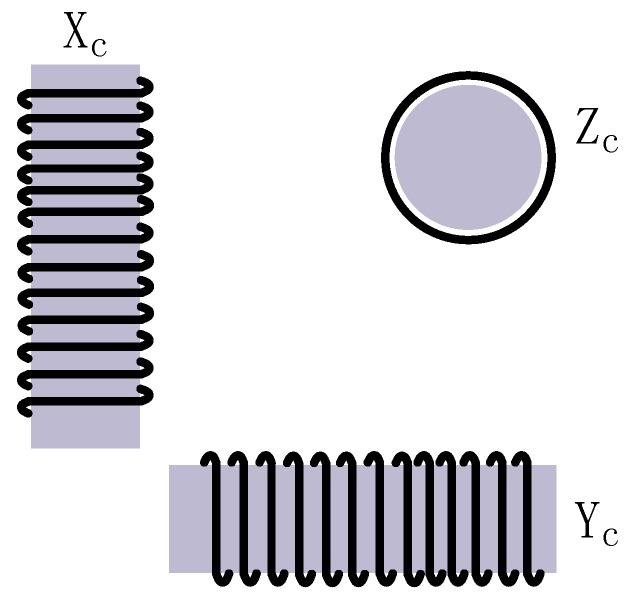
Coil layout of the magnetic induction sensor.

The basic construction of the sensor consists of three magnetic coils installed orthogonally to one another in three-dimensional space (Figure 1) that sense the carrier’s cutting the geomagnetic field in the rotation movement.

The three coils, X_c_, Y_c_, and Z_c_, have their normal vector pointing respectively to the positive direction of *x*-, *y*-, and *z*-axes, called the sensitive axes of the magnetic induction sensor. The coordinate origin is set on the flying body center of mass, with the three sensitive axes held in parallel with the three coordinate axes. The sensitive axes coincide, in theory, with the coordinate axes such that the components in the measurement carrier’s axial directions are measured to the highest accuracy. Nonetheless, in practice, some installation errors are unavoidable. Moreover, as the carrier cannot be solely made up of non-ferromagnetic materials, the magnetic field measured by the sensor would be the sum of the geomagnetic field and the one produced by ferromagnetic materials in the carrier. Additionally, redistribution of the geomagnetic field may result, and they constitute some sources of measurement error. In recognition of this, the following assumptions are made: first, the installation error is neglected in theoretical analysis; second, ferromagnetic material only affects the field within the flying body, its effect on the external field being neglected; third, the flying body is homogenous and axially symmetrical in both shape and mass.

### 2.2. Magnetic Field Response When the Magnetic Induction Sensor Rotates

The basic structures of the proposed magnetic induction sensor is its magnetic coils. Faraday’s law of electromagnetic law and the magnetic physics knowledge tell us that the magnetic flux variation within a closed coil that cuts a magnetic field will give rise to the following induced electromotive force (EMF):

(1) $$E=\frac{d\phi }{dt}=NBS\sin\omega t$$

where *N* is the number of turns, *B* the magnetic field strength responsible for coil induction, *S* the area enclosed by the coil, and ω the angular speed at which the coil rotates.

As can be known from the above equation, the sensor has a maximum EMF of

(2) E=NBSω.

We may look at the output of the magnetic induction sensor: *B* represents the local magnetic field, and when considering a short fly distance or short fly time, we may rightfully consider the geomagnetic strength to be constant; the area of the coil and the number of turns are also constant. Thus, the output of this type of sensor depends primarily on the rotation speed of the flying body.

A premium is put on miniaturized and highly sensitive sensors, so multiple layers of wound coil were used, with a coil area of 1 cm^2^, a wire diameter of 0.09 mm, and 9500 turns, and the coil resistance was 665 Ω. It is stated in the literature that, in Taiyuan (Shanxi Province in China), the geomagnetic strength is 0.5E−4T. It then follows that, at a rotation speed of up to 100 rps, the maximum EMF is 0.005 mV. Too many turns would give rise to noticeable magnetic leakage, greater noise, and lower SNRs, so a proper number of turns is desirable. Given that the rotational speed in practical applications varies in a wide range, it is beneficial to have a greater, yet adequate, number of turns and to amplify the output signals; otherwise, the output signal would be too low.

We may consider the induced voltage output by Coil X_c_. The original carrier coordinate system coincides with the geographic coordinate system. The flying body has in it a magnetic induction strength of *B*, whose components are $B_{gx}$, $B_{gy}$, $B_{gz}$ in the geographic coordinate system or $B_{x}$, $B_{y}$, $B_{z}$ in the carrier coordinate system, a magnetic declination of *D*, and a magnetic inclination of *I*. Then, in the geographic coordinate system the geomagnetic strength may be written as

(3) $$\left[\begin{array}{c} B_{gx}\\ B_{gy}\\ B_{gz} \end{array}\right]=\left[\begin{array}{c} -B\sin D\\ B(\mathrm{cosD}+\mathrm{cosI})\\ -B\sin I \end{array}\right].$$

The three coils having an identical effective area *S* as well as the number of turns *N*, according to Faraday’s law of electromagnetic induction, the magnetic flux of Coil X_c_, Y_c_, and Z_c_ is given by:

(4) $$\phi_{C}=\int_{SC}\vec{B}\cdot d{\vec{S}}_{C}=NBS\sin\omega t.$$

The magnitude of magnetic flux of Coil X_c_ in the case of single-axle rotation is as follows:

(1) When Coil X_c_ rotates at an angular speed of $\omega_{x}$ about the *X*-axis of the carrier coordinate system, $B_{x}$ is normal to Coil X_c_, so the flux through Coil X_c_ remains constant, with no induced voltage output; Coil X_c_ rotates about the *X*-axis and is in parallel with plane YOZ, and neither $B_{y}$ nor $B_{z}$ penetrate Coil X_c_, with no flux resulted, so Coil X_c_ has a magnetic flux:
  (5) $$\phi_{Cx1}=NSB_{x}.$$
(2) When Coil X_c_ rotates at an angular speed of $\omega_{y}$ about the *Y*-axis of the carrier coordinate system, in the initial position $B_{x}$ is normal to Coil X_c_, so the flux is maximum, and when the coil rotates to coincide with plane XOY the flux is 0; when $B_{y}$ is in parallel with X_c_, no flux results; in the initial position, $B_{z}$ is in parallel with Coil X_c_ and the magnetic flux is 0, and when Coil X_c_ rotates to coincide with XOY, the flux becomes maximum and Coil X_c_ a magnetic flux:
  (6) $$\phi_{Cx2}=NSB_{x}\cos\omega_{y}t+NSB_{z}\sin\omega_{y}t.$$
(3) When Coil X_c_ rotates at an angular speed of $\omega_{z}$ about the Z-axis of the carrier coordinate system, in the initial position, $B_{x}$ is normal to Coil X_c_, so the flux is maximum, and when the coil rotates to coincide with plane XOZ the flux is 0; in the initial position, $B_{y}$ is in parallel with X_c_ and no flux results, and when Coil X_c_ rotates to coincide with XOZ the flux becomes maximum; when $B_{z}$ is in parallel with Coil X_c_, the magnetic flux become zero and Coil X_c_ has a magnetic flux:
  (7) $$\phi_{Cx3}=NSB_{x}\cos\omega_{z}t+NSB_{y}\sin\omega_{z}t.$$

As is known from the above, in the movement process of the flying body, the instantaneous flux of Coil X_c_ is evaluated by

(8) $$\phi_{Cx}=NSB_{x}+NSB_{x}\cos\omega_{y}t+NSB_{z}\sin\omega_{y}t+NSB_{x}\cos\omega_{z}t+NSB_{y}\sin\omega_{z}t.$$

Similarly, in the movement process the instantaneous flux of Coil Y_c_ is

(9) $$\phi_{Cy}=NSB_{y}\cos\omega_{x}t+NSB_{z}\sin\omega_{x}t+NSB_{y}+NSB_{x}\sin\omega_{z}t+NSB_{y}\cos\omega_{z}t.$$

In the case of Coil Z_c_, its instantaneous magnetic flux in the movement process is

(10) $$\phi_{Cz}=NSB_{y}\sin\omega_{x}t+NSB_{z}\cos\omega_{x}t+NSB_{x}\sin\omega_{y}t+NSB_{z}\cos\omega_{y}t+NSB_{z}.$$

The EMF of Coil X_c_ is given by

(11) $$E_{Cx}=-\frac{d\phi_{Cx}}{dt}=NSB_{x}\omega_{y}\sin\omega_{y}t-NSB_{z}\omega_{y}\cos\omega_{y}t+NSB_{x}\omega_{z}\sin\omega_{z}t-NSB_{y}\omega_{z}\cos\omega_{z}t.$$

Similarly, the EMF of Coils Y_c_, Z_c_ is given, the EMF of Coils X_c_, Y_c_, Z_c_ is given by

(12) $$\left\{ \begin{array}{l} E_{Cx}=-\frac{d\phi_{Cx}}{dt}=NSB_{x}\omega_{y}\sin\omega_{y}t-NSB_{z}\omega_{y}\cos\omega_{y}t+NSB_{x}\omega_{z}\sin\omega_{z}t-NSB_{y}\omega_{z}\cos\omega_{z}t\\ E_{Cy}=-\frac{d\phi_{Cy}}{dt}=NSB_{y}\omega_{x}\sin\omega_{x}t-NSB_{z}\omega_{x}\cos\omega_{x}t-NSB_{x}\omega_{z}\cos\omega_{z}t+NSB_{y}\omega_{z}\sin\omega_{z}t\\ E_{Cz}=-\frac{d\phi_{Cx}}{dt}=-NSB_{y}\omega_{x}\cos\omega_{x}t+NSB_{z}\omega_{x}\sin\omega_{x}t-NSB_{x}\omega_{y}\cos\omega_{y}t+NSB_{z}\omega_{y}\sin\omega_{y}t \end{array}\right..$$

Equation (12) constitutes the movement magnetic response equation set for the magnetic induction sensor; particularly, they contain the angular speed data of the carrier’s three axes.

## 3. Design of the Electronic Solver Module

In measuring the angular speed of a rotating body, it is necessary to adapt to the frequency variation effect of the sensor response’s physical quantity, in particular when such periodic signal frequency varies quickly, in which case the angular speed measurement accuracy tends to deteriorate. There is still another problem: for the same signal in the same period and after A/D conversion, the phase value of the low-frequency output is greater than that of the high frequency, particularly in the response where the signal rises sharply and then falls instantaneously. Another need is to implement direct phase measurement by non-program algorithm processing. To address the above challenges, the proposed self-adaptive phase measurement system uses a strategy that combines analogue signal processing with FPGA control.

**Figure 2:**
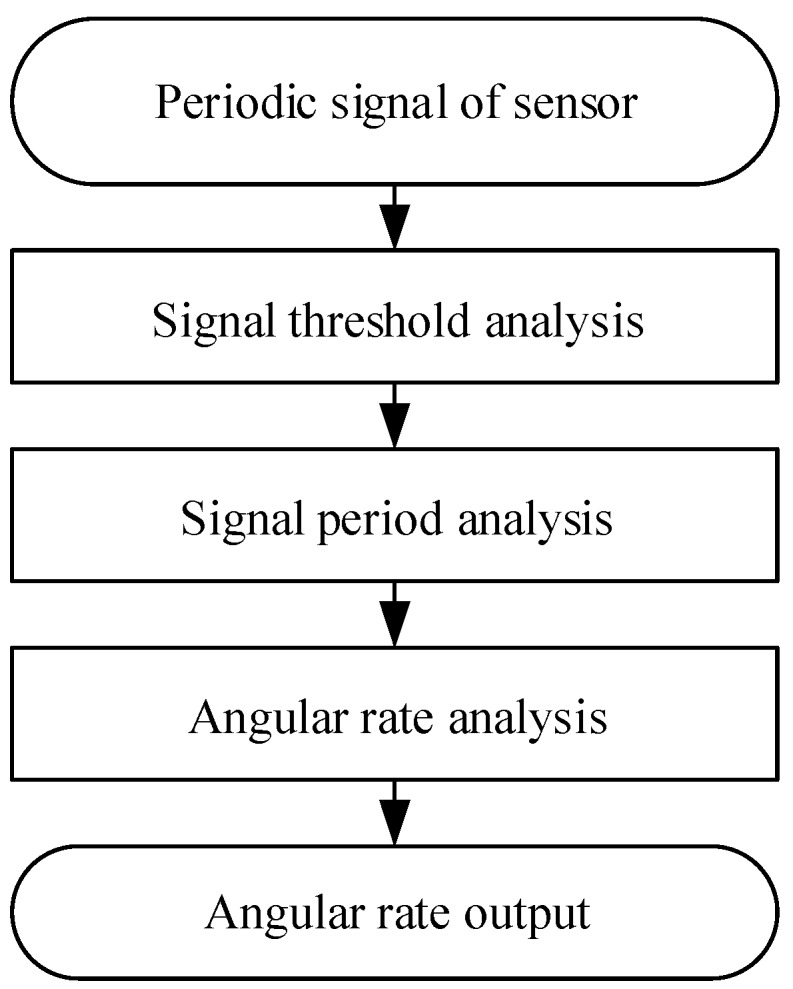
Composition of the angular rate electronic solver module.

The solver module consists of three major parts: signal processing, signal period analysis, and angular rate analysis. Its block diagram is shown in Figure 2. The sensor signal processing part is designed to pre-process sensor output signal and, with recourse to positive and negative threshold analysis, to find the “zero” phase point. The signal period analysis part is responsible for signal period analysis based on the “zero” phase and then determines the signal period. The angular rate analysis part analyzes periodic signal to count the “zero” phases, and the counting is then converted into the angular rate.

### 3.1. Threshold Analysis of the Sensor Signal

**Figure 3:**
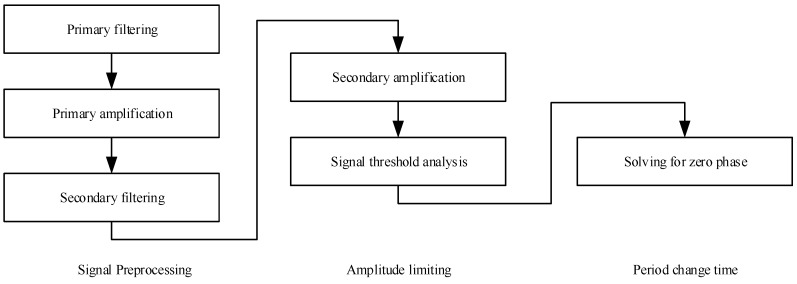
Flow process of electronic signal threshold analysis.

Threshold analysis of the sensor signal has a process flow as shown in Figure 3. The signal output directly from the sensor contains noises of various frequencies. Primary filtering, which is performed depending on different types of physical quantities, is useful in reducing physical background high-frequency interference signals and improving the sensor SNR. After primary filtering, the sensor signal strength is low, on the order of mV, and subsequent circuits may have difficulty detecting the signal. Hence, the signal after primary filtering needs primary amplification. The signal, when amplified, goes through secondary filtering, whereby the amplified signal is processed by a low-pass filter such that the low-frequency signal is retained and burr noises are removed. Secondary amplification is then carried out, this time using automatic gain to control signal strength, such that the strength is maintained within a certain range; this saves linearly amplified signal from being truncated when the signal strength varies in a large range.

The signal having been secondarily amplified is then studied in threshold analysis, which involves comparison against positive or negative thresholds. A signal greater than “zero” is compared against the positive threshold, a signal below “zero” is compared against the negative threshold, and a signal falling between positive and negative thresholds are considered noise. What comes next is solving for the zero phase. A zero phase is believed to exist when the signal crosses the negative and the positive thresholds consecutively, and the midpoint between the two thresholds is taken to be the zero phase point.

### 3.2. Period Analysis of the Sensor Signal

Threshold analysis enables us to determine the time corresponding to the zero phase of the output periodic signal, or the start point of the periodic signal, by means of which the latch of count value is controlled; then, the counter zero is reset and the counting starts again.

Once the counter is put into work, the first zero-crossing signal is taken as the start point t_0_ of the first period, and the second one serves as the start point t_1_ of steady periods, symbolizing the begin of zero phase counting, and each subsequent zero-crossing t_i_ then acts as the half-period time interval of the signal.

Sensor signal depends for its period accuracy on the sampling circuit accuracy. In period analysis, signal filtering and delay adjustment are performed to reduce zero-crossing misjudgment probability.

### 3.3. Angular Rate Analysis of the Sensor Signal

**Figure 4:**
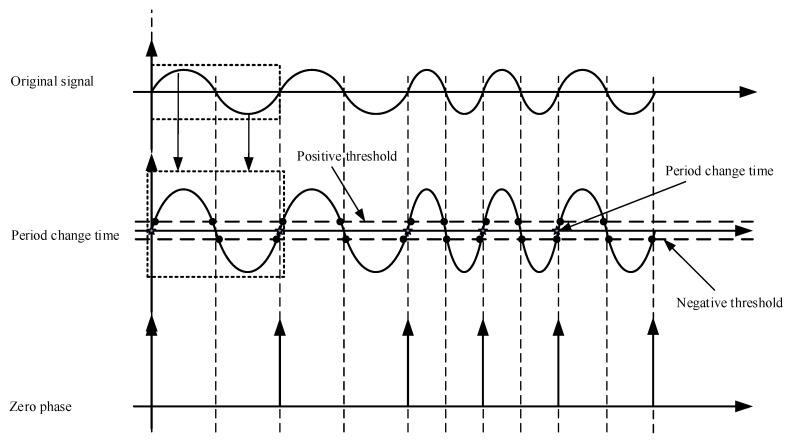
Conversion process of the coil sensor zero phase signal.

The conversion of a sensor signal is a process as shown in Figure 4. The original signal is the raw signal output of the sensor. From the original signal changing at the shown frequency, the period change times are found after threshold analysis by the analogue circuit; the analogue comparator by means of signal period analysis establishes a zero phase (which appears at time t_j_); the zero phase voltage plays a role as an angular rate resetting bit, controlling the recounting of the angular rate.

(13) $$\omega =(t_{j+1}-t_{j})/2$$

where j is 0, 1, 2, 3, ...

In the rotation process of the sensor, induced EMF are created on the three orthogonal coils, like the voltage output of a generator, called the “original signal” in Figure 4. This signal is fed into the “electronic solver module,” which analyzes the signal against the preset signal thresholds, or the “positive” and “negative” thresholds as shown in Figure 4. If the input signal varies between the positive and negative thresholds, the solver module regards it as noise and in this case no zero phase is output; in other words, no such “period change point,” as shown in Figure 4, is output. If the input signal exceeds either the positive or the negative threshold, the solver likewise treats it as noise. Only when the input signal crosses the negative and then the positive threshold, or vice versa, will the solver accept the signal as a valid period change point. When the signal crosses for the first time the negative threshold and then crosses the negative threshold, the solver decides that this period change point qualifies as a zero phase. When the signal crosses the negative threshold and then crosses the positive threshold for the second time, the solver knows that this signal signifies the end of this period; this period change point is not only the end of this period but also the beginning of the next period, i.e., a zero phase of the new rotation period.

Zero phases as found are substituted into Equation (13) to get the angular rate corresponding to this moment.

## 4. Experimental Results and Discussion

### 4.1. Semi-Physical Simulation Test

**Figure 5:**
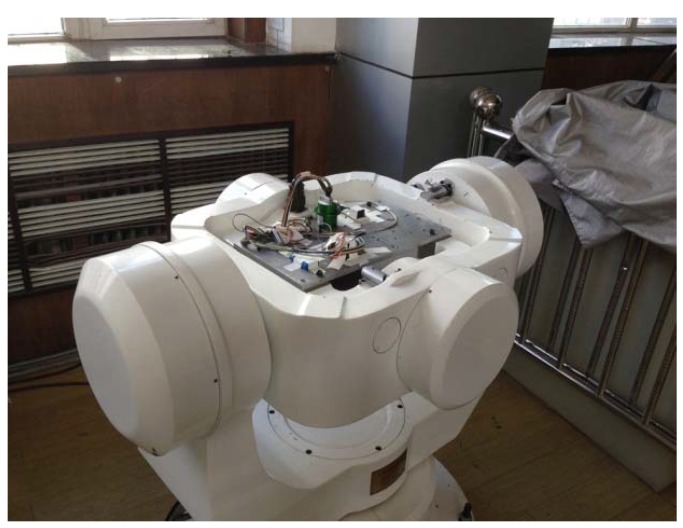
Three-axle rotary table layout of the magnetic induction angular rate sensor.

For testing, a magnetic sensor was installed in an analogue rotating magnetic field; see Figure 5. The sensor generated a sinusoidal signal that changes periodically, and the signal was analyzed for threshold as described in Figure 4. A semi-physical high rotational speed simulation was performed with the sensor fixed on a three-axle flying simulation rotary table. The sensor output voltage values, as measured in single-axle high-speed rotation, were used to provide the solution, and the results were compared with the preset rotation speed of the table to determine the sensor angular rate accuracy.

**Figure 6:**
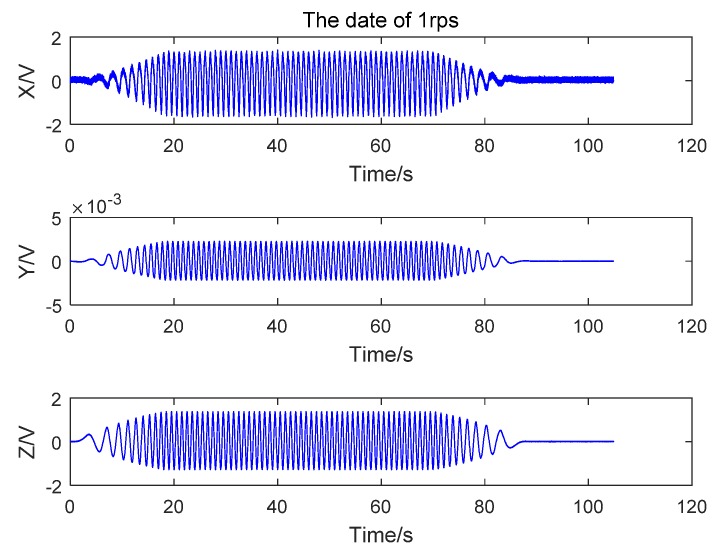
Sensor induction voltage when the rotary table intermediate coil rotation rate is 1 rps.

The sensor was mounted on the inner frame of the three-axle flying simulation rotary table, which was started after its rotation speed had been set. In responded to the rotation, the sensor sent out a signal. The inner coil of the rotary table accelerated at a constant acceleration rate of 20°/s until reaching an angular rotation speed of 360°/s. The output values of the three axes are given in Figure 6. Specifically, the output voltages of the *x*- and *y*-axes are a function of *ω_z_*, but the output corresponding to the *y*-axis should be zero. The actuality is nevertheless that the *y*-axis has voltage output due to table vibration and sensor installation error.

**Figure 7:**
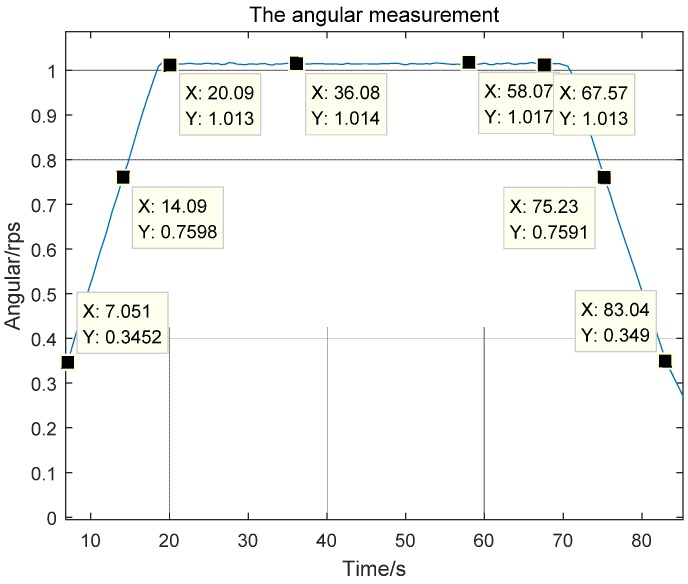
Instantaneous intermediate coil angular speed output by the magnetic sensor electronic solver module.

As is apparent from Figure 7, the sensor read an angular rate of 1.013–1.018 rps when the rotary table stabilized at 1 rps, producing an error of 1.3‰–1.8‰ FS; the initial angular rate measurement error was greater, which is explained by unstable measurement of zero-crossing signal at the initial stage.

### 4.2. Physical Test of Hi-Precision Rotary Table

**Figure 8:**
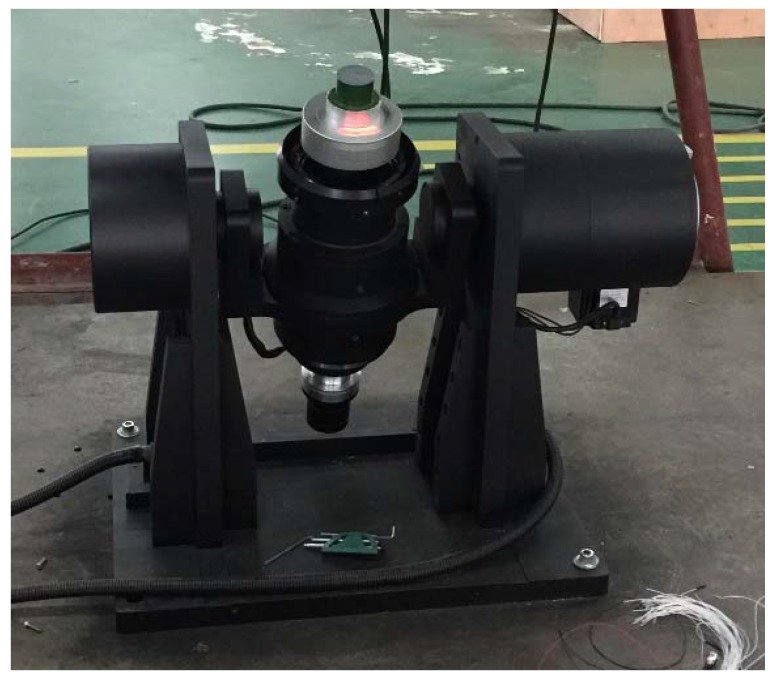
Single-axle rotary table layout of magnetic induction angular rate sensor.

The sensor was mounted on a single-axle hi-speed rotary table, which was started with an angular speed set at 100 rps. The table cannot be accelerated to its set value in a single stop, so this was done in three steps. At the end of each acceleration, the machine was given time to stabilize down before the next acceleration, until the set 100 rps was reached. The physical testing layout of the magnetic induction sensor is shown in Figure 8.

**Figure 9:**
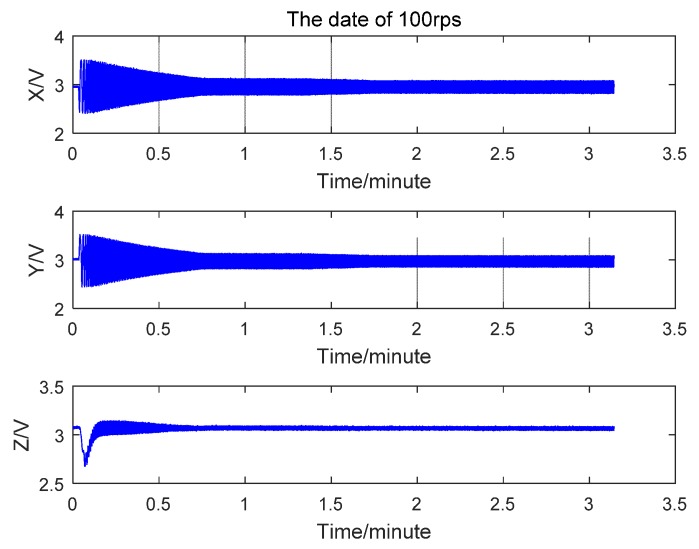
Sensor induction voltage when the single-axle rotary table rate was 100 rps.

In response to the hi-speed rotation of the table, the three axial coils generated induced voltages as shown in Figure 9. The *z*-axis ran in parallel with the table rotary axle, and in this rotation the coil effective projection cross area was less than the coil cross area, so the *z*-axis produced an induction voltage with a minimal peak.

**Figure 10:**
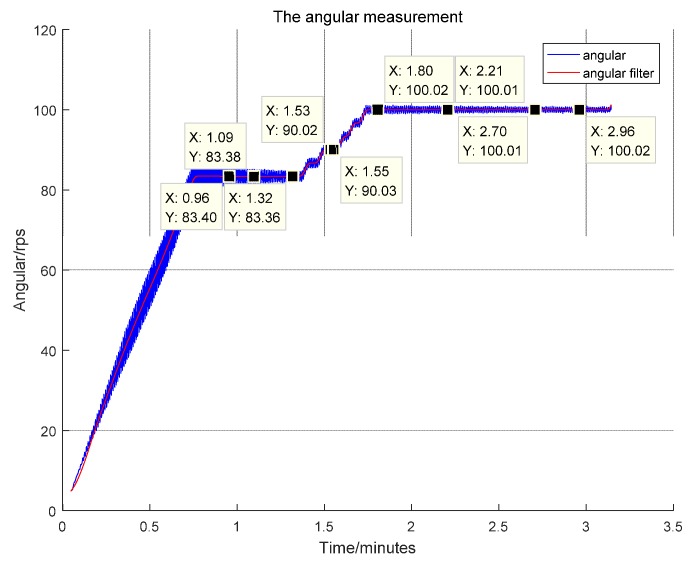
Instantaneous single-axle angular speed output by the magnetic sensor electronic solver module.

In simulation, the hi-speed rotary table is accelerated to the preset 100 rps in three steps. In the first step, the rate is accelerated to 83.3 rps, as illustrated in Figure 10 and the table is allowed to stabilize down over time. Then, the rate is accelerated to 90 rps, and the angular rate is measured once again; when the table stabilizes at 90 rps, the table is set to 100 rps this time, and the rotational rate is measured in real time using the sensor.

After analysis of the measurement data, the sensor’s output is found, as shown in Figure 10, by use of the angular rate solver module and the signal analysis process as given in Figure 4. In the three-step acceleration course, the sensor registered 83.36–83.40 rps when the rotary table rotated at 83.3 rps, producing an error of 0.7‰–1.2‰ FS. The sensor read 90.02–90.03 rps when the table rotated at 90 rps, giving an error of 0.2‰–0.3‰ FS. The sensor recorded 100.01–100.02 rps when the table rotated at 100 rps, involving an error of 0.1‰–0.3‰ FS.

### 4.3. Sensor Performance Analysis

The sets of data acquired by the sensor in low-speed and hi-speed rotary table simulations are tabulated in Table 1.

A study of the sensor’s adaptability to measure discrete angular rates finds that the sensor for hi-speed rotating angular rate measurement is better. This is explained by the fact that the coil EMF increases in rotation angular rate. The induced EMF are forwarded into the solver module for a “periodic signal time” analysis, and the higher the EMF, the higher the signal SNRs will be, and the lower the error in the determination of periodic signal points will be.

**Figure 11:**
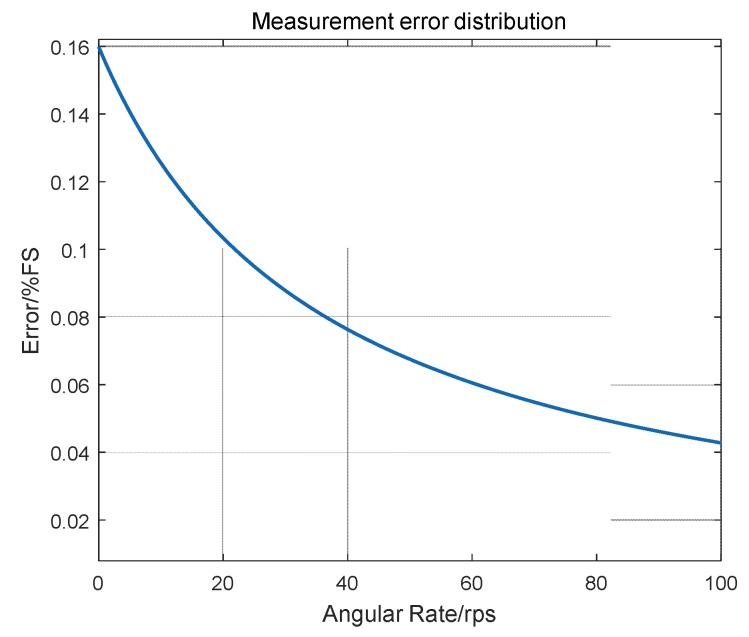
Sensor measurement error vs. rate.

A measurement error analysis of the sensor was conducted, and Figure 11 shows the sensor measurement error as a function of the angular rate. As can be seen from Table 1 and Figure 11, the proposed sensor performs better when measuring hi-speed angular rates.

The above tests demonstrate that the proposed sensor is capable of measuring angular rates of low-speed and hi-speed rotation bodies as well as pinpointing the zero-phase time of the output physical quantity. With such periodic points, it is possible to reset the start points of periods, which is useful for improving the sensor’s measurement performance.

## 5. Conclusions

A new type of magnetic induction rotation angular rate sensor is proposed in this paper to satisfy the industrial needs for wide-range measurement of rotational angular rate. A model is created that represents the coil layout of the sensor. This paper includes a discussion of the magnetic induction behavior of coils moving in a magnetic field, and based on this discussion a model is developed to describe the physical response of the magnetic induction sensor carrier in a rotary movement. Magnetic induction signal characteristics are investigated, and, based on the periodicity of magnetic induction signal, an electronic solver module is built for the sensor. When tested on a low-speed rotary table at a 1 rps angular rate, the sensor scored a measurement error of 1.8‰ FS. When tested on a hi-speed rotary table at a 100 rps angular rate, the sensor registered a measurement error of 0.2‰ FS. The test results suggest that the proposed sensor can be used in internal single-axle measurement applications where the rotation rate ranges from 1 to 100 rps, with better measurement accuracy at higher rotation rates.

## Figures and Tables

**Table 1:** Measurement errors of the magnetic induction sensor.

Angular Rate Setting (rps)	Maximum Read-out (rps)	Minimum Read-out (rps)	Maximum Error (‰ FS)	Maximum Error (‰ FS)
1	1.018	1.013	1.8	1.3
83.3	83.40	83.36	1.2	0.7
90	90.03	90.02	0.3	0.2
100	100.02	100.01	0.2	0.1
